# EAAS AND WHEY PROTEIN MAY IMPROVE PHYSICAL FUNCTION OF OLDER ADULTS INDEPENDENTLY OF GUT MICROBIOME

**DOI:** 10.1093/geroni/igad104.2996

**Published:** 2023-12-21

**Authors:** Gohar Azhar, Ambika Verma, Intawat Nookaew, Pankaj Patyal, Shakshi Sharma, Robert Wolfe, Jeanne Wei

**Affiliations:** University of Arkansas for Medical Sciences, Little Rock, Arkansas, United States; University of Arkansas for Medical Sciences, Little Rock, Arkansas, United States; University of Arkansas for Medical Sciences, Little Rock, Arkansas, United States; University of Arkansas for Medical Sciences, Little Rock, Arkansas, United States; University of Arkansas for Medical Sciences, Little Rock, Arkansas, United States; University of Arkansas for Medical Sciences, Little Rock, Arkansas, United States; University of Arkansas for Medical Sciences, Little Rock, Arkansas, United States

## Abstract

Essential amino acids (EAAs) and dietary proteins are major nutritional supplements that support the growth and activity of gut microbes, contributing to the wellbeing of their host. We hypothesized that daily ingestion of EAAs or whey proteins for twelve weeks would improve the gut microbiome of older adults and thereby have a positive impact on their physical functioning. The stool samples were collected from the 40 subjects before and after consuming EAAs or whey proteins for twelve weeks and subjected to Illumina based 16S rRNA gene sequencing. In both groups, the most abundant families in order of relative abundance included: Bacteroidaceae, Ruminococcaceae, Lachnospiraceae, Rikenellaceae, and Prevotellaceae, which indicated that these subjects were able to maintain a dominant consortium of microbes in their guts that have been associated with healthy aging. Individual gut microbiome samples analysis with nonmetric multidimensional space (NMDS) showed that each individual was associated with its unique microbiome. The key finding of this study was a significant reduction of plasma Interleukin-18 binding protein (IL-18) in EAAs vs whey group (p < 0.003). It is plausible that the reduced level of inflammatory IL-18 contributed to improved intestinal immunity. Moreover, the reduced inflammation might have resulted in better muscle performance in the EAAs group vs whey as evidenced by six-minute walking distance (EAAs 92.55 ± 4.16 ft vs whey 47.46 ± 4.99 ft, p< 0.0001) at the completion of study. Future studies will need to explore the role of EAAs on gut microbiome mediated reduction of immune inflammation on physical function and aging.

